# Impact of Chronic Oral Administration of Gold Nanoparticles on Cognitive Abilities of Mice

**DOI:** 10.3390/ijms24108962

**Published:** 2023-05-18

**Authors:** Alexandra L. Ivlieva, Elena N. Petritskaya, Dmitriy A. Rogatkin, Inga Zinicovscaia, Nikita Yushin, Dmitrii Grozdov

**Affiliations:** 1 Institute of Higher Nervous Activity and Neurophysiology of RAS, 5A Butlerova St., 117485 Moscow, Russia; ivlieva@medphyslab.com (A.L.I.); medphys@monikiweb.ru (E.N.P.); rogatkin@medphyslab.com (D.A.R.); 2 Moscow Regional Research and Clinical Institute named after M. F. Vladimirsky, Str. Schepkina 61/2, 129110 Moscow, Russia; 3 Joint Institute for Nuclear Research, Str. Joliot-Curie 6, 141980 Dubna, Russia; ynik_62@mail.ru (N.Y.); dsgrozdov@rambler.ru (D.G.); 4 Horia Hulubei National Institute for R&D in Physics and Nuclear Engineering, 30 Reactorului Str., MG-6, RO-76900 Bucharest-Magurele, Romania; 5 Institute of Chemistry, Academiei Str. 3, MD-2028 Chisinau, Moldova

**Keywords:** gold nanoparticles, neutron activation analysis, anxiety, cognitive functions, development

## Abstract

The influence of gold nanoparticles after their prolonged oral administration to mice (during pregnancy and lactation) on spatial memory and anxiety levels in offspring was investigated. Offspring were tested in the Morris water maze and in the elevated Plus-maze. The average specific mass content of gold which crossed the blood–brain barrier was measured using neutron activation analysis and constituted 3.8 ng/g for females and 1.1 ng/g for offspring. Experimental offspring showed no differences in spatial orientation and memory compared to the control, while their anxiety levels increased. Gold nanoparticles influenced the emotional state of mice exposed to nanoparticles during prenatal and early postnatal development, but not their cognitive abilities.

## 1. Introduction

For each new and potentially widely used medical compound, the question about its safety for patients, especially for pregnant women and children arises. Gold nanoparticles (AuNPs) already found wide applications in medicine—in optical visualization, target photosensitization, as sensitizers in radiotherapy of tumors, and as carriers for different drug molecules (antioxidants, immunomodulators, etc.) [1,2,3,4,5,6]. The ability of AuNPs to cause oxidative stress reported in several studies [7,8] allows their applications as sensitizers [9]. Because of the chemical inertness of gold [10], AuNPs are among the least toxic nanoparticles for mammals [3].

However, in several studies, it was shown that AuNPs are able to penetrate the blood–brain barrier and accumulate in the brain [8,11,12,13]. They can cause oxidative stress in neurons and glial cells [7,8,14], and increase expression of the markers of inflammation in the brain [11]. The ability of AuNPs to bind to DNA was shown in [15,16]. As it was detected applying the methods for estimation of genotoxicity samples treatment with AuNPs increases the damage of DNA, both, in in vitro and in vivo studies [17]. The ability of small nanoparticles to cross biological barriers is one of their most dangerous properties, despite their usefulness for different medical applications, especially for brain disease treatment [18]. High penetration ability makes not only the brain but also gonads, fetus (through the blood–placenta barrier) [19], and infants (through the milk of mother who had contact with AuNPs) vulnerable to AuNPs’ impact.

It should be noted that the behavioral effects caused by contact with AuNPs are described in only several studies, mainly for adult animals after single or several injections [13,20]. Therefore, the present study aimed to examine the influence of AuNPs administrated to mice during pregnancy and lactation on spatial orientation, memory, and anxiety levels in offspring. The accumulation of gold in different tissues of female mice and their offspring was assessed using neutron activation analysis. To our knowledge, it is the first study in which the prolonged effect of the AuNPs on the offspring was studied.

## 2. Results

### 2.1. Distribution of Gold in the Organs and Tissues

After oral intake, AuNPs were transported to different organs via the bloodstream. It should be mentioned that the content of gold in the blood of females and offspring was lower than in analyzed organs (Table 1). A slightly higher content of gold was detected in mothers’ blood compared to offspring’. In control mice, the content of gold in all analyzed organs was below the detection threshold of the NAA technique.

Accumulation of gold in different organs and blood was similar in mothers and offspring: the lowest content was accumulated in the brain (Table 2), then, ten times higher, in blood, followed by lungs, liver, and the highest—in kidneys (Table 1). The content of gold in the liver and kidneys of offspring was ten and two times, respectively lower than in females.

### 2.2. Elevated Plus-Maze Results

All mice (SHK line, 20 experimental, 10 control animals) spent most of the trial time in the closed arms, less time—in the center, and visited the open arms rarely or not at all (except one experimental animal which sat in the open arm longer than other animals) (Supplementary Material). In comparison with control mice, experimental animals run more rarely (number of events (running) *p* = 0.008), but longer (mean length (running) *p* = 0.043). These mice entered the closed arm quicker (latency to the first event (closed arms) *p* = 0.022), run into the arm’s end, and sat there longer (summary length (closed arms) *p* = 0.18, mean length (closed arms) *p* = 0.019). In addition, experimental animals more rarely run from one arm to another (number of events (closed arms) *p* = 0.015, number of events (center) *p* = 0.014, summary length (center) *p* = 0.002), and less frequently looked into the center while sitting in the arm (summary length (stretch-attend posture) *p* = 0.023, mean length (stretch-attend posture) *p* = 0.009). Experimental mice groomed less often, but longer (mean length (grooming) *p* = 0.004) and reared longer and more frequently (mean length (rearing) *p* = 0.0003). A similar pattern was observed when only males (10) or females (10) of the experimental group were compared with control mice.

### 2.3. MWM Results

**Figure 1:**
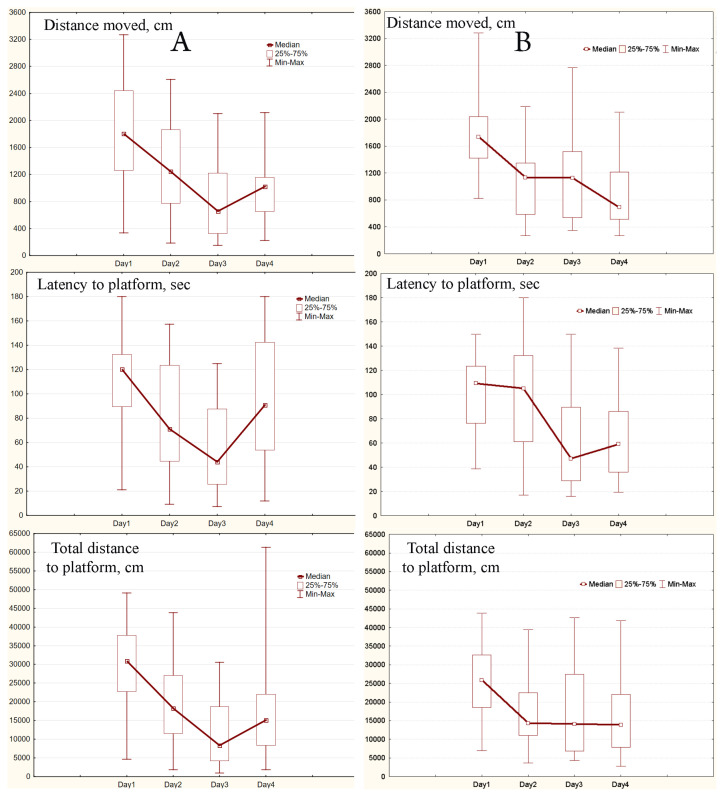
Learning curves obtained for studied groups of animals: (**A**), experimental mice (SHK line, 24 animals), (**B**), control mice (ICR and SHK lines, 18 animals). The central point represents the median; boxes, the lower and upper quartiles; intervals, the ranges.

**Figure 2:**
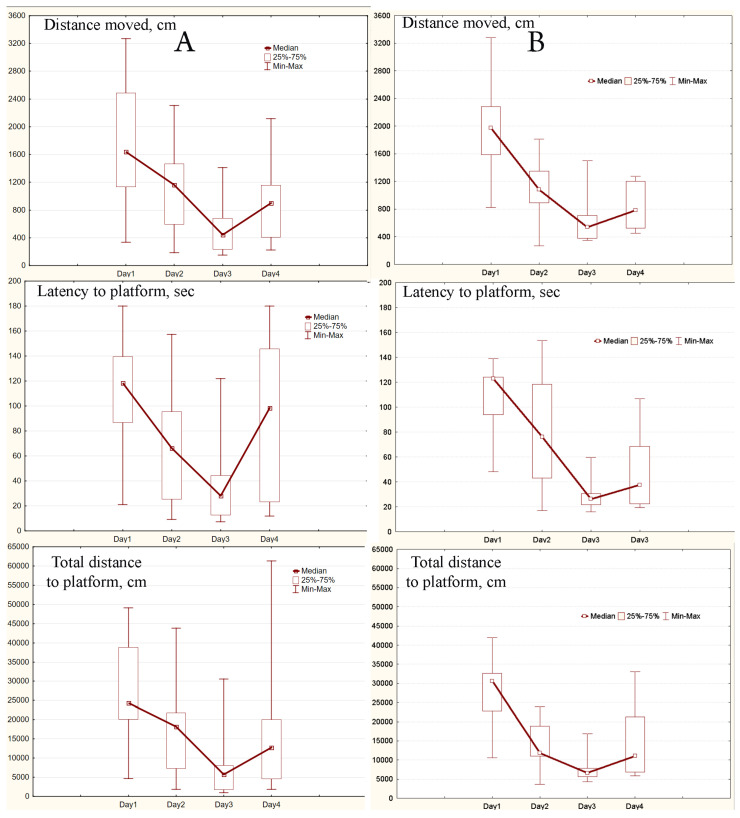
Learning curves obtained in the capable group: (**A**), experimental mice (SHK line, 15 animals), (**B**), control mice (ICR and SHK lines, 9 animals). The central point represents the median; boxes, the lower and upper quartiles; intervals, the ranges.

**Figure 3:**
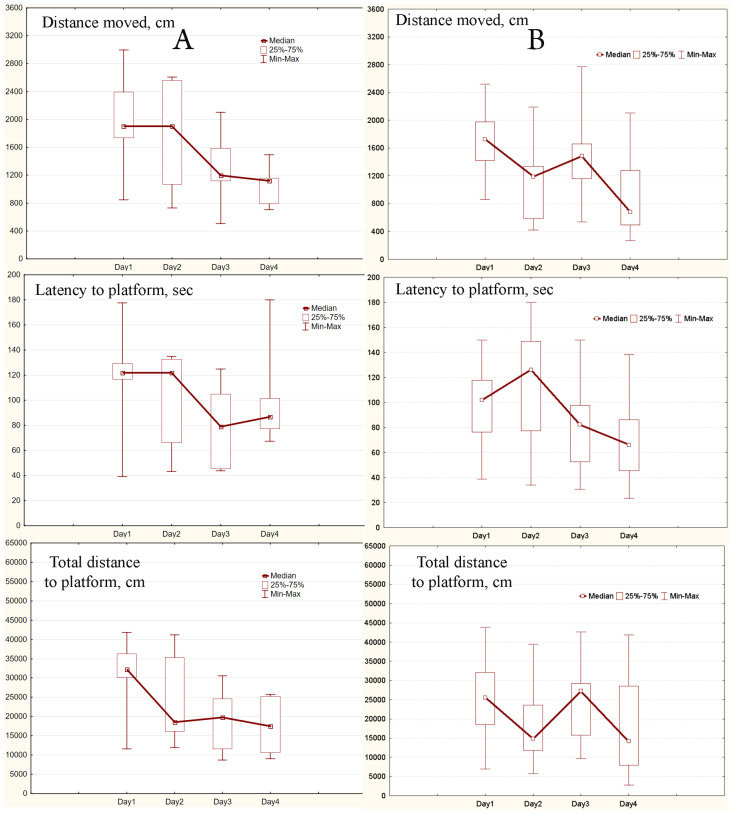
Learning curves obtained in the intermediate group: (**A**), experimental mice (SHK line, 9 animals), (**B**), control mice (ICR and SHK lines, 9 animals). The central point represents the median; boxes, the lower and upper quartiles; intervals, the ranges.

After three days the learning curves typical for MWM were obtained for experimental and control mice and each group. As it can be seen the daily medians and means of all three parameters were declining (Figure 1, Figure 2 and Figure 3, Tables S1–S6). Values of the parameters from Day 1 and Day 3 were significantly different for all three parameters (all *p* < 0.0033), both in experimental and control mice. Similar significant differences were found in capable and intermediate experimental animals (all *p* < 0.039). However, in control mice differences were significant only in the capable group (all *p* < 0.0077). 

After a change of platform position in the pool on Day 4 in capable experimental mice parameters’ values significantly increased compared to Day 3 (all *p* < 0.013). At the same time, in intermediate experimental mice and in control one they did not change or slightly decrease (Figure 2 and Figure 3). In pairwise comparison between daily values of parameters for experimental and control animals the only differences, the total distance to the platform in the whole sample and the intermediate mice (*p* = 0.038 and *p* = 0.034, respectively), were observed on Day 3.

## 3. Discussion

A significant amount of gold was determined in all tissues of experimental mice and their offspring. The latter contradicts the data that were obtained after acute contact of animals with bare or citrate-coated AuNPs introduced by several intravenous injections to pregnant mice [21]. No traces of gold were found in tissue sections of fetuses (and placenta) by TEM [22], while ICP-MS did not detect gold in fetal organs, and only in significant amount in placenta [21]. It may indicate a slow rate of AuNPs transfers through the blood–placenta barrier. In the abovementioned studies, tissues were taken 24 h after two- or three-day injections. Thus, it can be suggested that AuNPs were not transferred to the fetus 3–4 days after the first dose.

The low content of gold in the blood of experimental animals indicates the rapid transfer of AuNPs from blood to different organs, which confirms the high permeability of tissues for very small (<20 nm) nanoparticles [18]. The gold content in mothers’ blood and liver was higher than in offspring, which is consistent with the assumption about the slow rate of AuNPs’ transfer through the blood-placenta barrier. This finding contradicts the data obtained in a similar experiment with silver nanoparticles, where the amount of silver in the offspring’s blood was four times higher than in females [23]. 

The highest content of gold was determined in the kidneys of both females and offspring. In [24] it was shown that after 28 days of daily intraperitoneal injections of PEG-coated AuNPs with the size of 5 and 10 nm in adult mice, the highest amounts of gold were accumulated in the liver and spleen. It should be noted that the predominant accumulation of gold in the liver and spleen was detected 24 h after the single intravenous injection of 20 nm sized citrate-coated AuNPs [12], and after 8 days of daily intraperitoneal injections of 12.5 nm sized citrate-coated AuNPs [25]. Although in the latter study, the gold content in the kidneys was higher than in the spleen. The total daily intake of AuNPs per animal in [4] was comparable with the present study, and while the content of gold in kidneys was close to whose determined in the present work, in the liver its content was eight times higher. Although in study [4] the high accumulation of gold in organs can be explained by direct injection of a whole daily dose, the comparatively low amount of gold in the mother’s liver in the present study is consistent with considerable amounts of gold found in offspring. It is suggested that nanoparticles were transferred to offspring instead of accumulating in the mother’s liver. Thus, it can be assumed that the excretion of AuNPs was mostly done by kidneys, while the liver eliminated AuNPs from the bloodstream and stored them, although it could not prevent AuNPs’ transfer to the fetus and milk. The opposite results were obtained for silver nanoparticles in [23], the content of silver in kidneys was the lowest compared to other organs. 

The content of gold in the liver was the second largest in the present study and the highest in [4,12]. The liver has a high density of blood vessels, and the phagocytosis of nanoparticles, including citrate- or PEG-coated AuNPs, by Kupffer cells after acute contact through intravenous (quick uptake) or intraperitoneal (slower uptake) injection was described [22,26,27]. It should be noted that macrophages in other tissues (in mesenteric lymph nodes, spleen, and small intestine) also accumulated AuNPs, but in smaller amounts than liver Kupffer cells [22]. The high content of gold was also determined in the spleen [4,12,27]. However, the content of gold in the offspring’s liver was lower than in the liver of females, and it can correlate with the lower content of gold in the offspring’s blood. In contrast, the silver content in the liver did not differ between mothers and offspring [23]. Lungs have very dense vascularization, and it can be suggested that gold content in the lungs most likely correlates with the content of the metal in blood. However, in [25], the gold content in the lungs of adult mice was comparable with the present study, even though the total daily intake of AuNPs was approximately ten times lower.

After long-term exposure, the accumulation of gold in the brain was the lowest both in females and offspring, which is in line with the data obtained after 24 h [12] and 8 days [25] for adult mice exposure to AuNPs. The known relative inertness of AuNPs, especially in contrast to the high chemical activity of nanosilver [10], could be responsible for the low accumulation of AuNPs in the brain despite the high permeability of the blood–brain barrier for very small nanoparticles. 

In an elevated plus-shaped maze all young mice demonstrated a preference for dark and secluded places over open areas, which is typical for rodents. Compared with control animals, experimental mice demonstrated signs of higher levels of anxiety: prolonged stays in closed arms with accompanying increase in long grooming, and decreased running. The exploratory behavior of experimental mice was unevenly changed: stretch-attend posture was shorted, but rearing with support was increased. It could be related to the amount of body exposure to open spaces that were necessary to perform both elements of behavior: for rearing it was enough to expose the nose and part of the head, while the whole half of the body was in the open arm in stretch-attend posture. Thus, the exploratory behavior of experimental animals could not be reduced as a whole but adapted for better body shielding, which is consistent with heightened anxiety. These data contradict the results on the estimation of anxiety levels in an open field test that was done on adult mice after several consecutive injections of bare or PEG-coated AuNPs [13], where no differences were found in comparison to control animals. Such discrepancies could be influenced by the difference in the design of the two tests, as an open field does not have a dark hiding place. A similar pattern was observed in the experiments in which the influence of silver nanoparticles on anxiety levels in offspring was estimated [28,29]. It can also indicate increased vulnerability of the developing brain to AuNPs’ effects. Additionally, the coating could influence the anxiety levels by enabling different mechanisms of AuNPs’ accumulation in brain. In both tests, open field and elevated plus-shaped maze, intraperitoneal injection of glycoprotein transferrin coated AuNPs to the adult mice enabled the carrier-mediated transport through the blood–brain barrier and demonstrated increased anxiety (without changes in locomotion). It should be mentioned that after similar contact with bare AuNPs, no changes in anxiety were detected [20]. 

In the Morris water maze, no signs of impairments in spatial orientation and memory were found; animals successfully learned to find the platform. The differences in parameters’ dynamic on Day 4 between experimental capable and intermediate mice correlate with the following types of behavior characteristic for these groups: capable animals with directed search or “scanning” were initially confused, which had raised the parameters’ values, but for intermediate mice with random search, the probability of finding the platform did not change or slightly increased. The only difference between experimental and control animals in pairwise comparison was the total distance to the platform on Day 3, which can be attributed to the different types of behavior, which allowed capable animals to find the platform quicker. It should be noted that the small number of the control sample of capable and intermediate mice influenced the statistical significance of comparisons in the control group, but the parameter’s dynamic typical for successful learning was clearly shown in the control samples. The lack of differences between experimental and control mice agrees with the data reported in [30] for adult mice after a single injection of citrate-coated AuNPs. The lack of deficiencies in spatial learning in the Barns maze after regular intraperitoneal injections of citrate-coated AuNPs to adult mice was shown in [31].

## 4. Materials and Methods

### 4.1. Animals

Outbred white mice (SHK) of the age 1.5–2 months (with an average mass of 20 g), 15 females and 5 males, were purchased from the Stolbovaya Farm (Moscow region, Russia). Mice are heterozygous by an undefined number of genes and are used to assess the safety of medical and cosmetic products, and dietary supplements. The animals and their offspring were maintained in the vivarium of M.F. Vladimirskiy Moscow Regional Research and Clinical Institute. Females were kept in steel cages with sizes of 31.5 × 23 × 15.7 cm, and each cage contained five mice, with a natural cycle of illumination (by daylight through the window in the period from 8 am to 5 pm daily) and the temperature of 22–24 °C; each cage was cleaned once per day. The methodology of the experiments and the maintenance of the animals in the vivarium of the institute were performed according to the principles of Directive 2010/63/EU of the European Parliament and of the Council of 22 September 2010 on the protection of animals used for scientific purposes [32].

### 4.2. Nanoparticles

Small, 3–5 nm in diameter, AuNPs were selected since the accumulation of smaller particles is usually higher and more widespread than that of larger ones [18]. High-purity polyethylene-glycol-coated gold nanoparticles (PEG-AuNPs) were purchased from the M9 Company (Tolyatti, Russia). According to the manufacturer, the nanoparticle solution is stable for two months. The experimental solution with a concentration of 25 μg/mL was prepared by diluting the concentrated solution with pure water in a ratio of 1:500. The bottles in cages were filled with the solution when its level was low, but at least once a week. The experimental solution was prepared anew every two weeks.

### 4.3. Experiment

To study the uptake of AuNPs by both mothers and by offspring, experimental females drank the experimental solution with a concentration of 25 μg/mL a week before pregnancy and to the end of lactation (1 month after birth). The total daily intake per animal was 125 μg—the daily liquid consumption per mouse was 5 mL [33]. Thus, the offspring of experimental females received AuNPs from mothers—both through the placental barrier during prenatal development and with milk during lactation. Data for control females and their offspring were taken from the similar experiment performed previously with silver nanoparticles [23]. After the end of the lactation, young experimental animals, 15 females and 15 males, were transferred to their own cages, 3 mice per cage, where they drank tap water and were raised to 2 months of age. Then, animals participated in behavioral tests.

### 4.4. Measuring of Gold Content

At the end of the lactation, females and some of the offspring were euthanized by intraperitoneal injection of urethane solution. The concentration of the water solution is based on body weight and a dosage of 1.2 g of dry urethane per kg of body weight, proposed by a veterinarian. Brain, liver, lungs, kidneys, and blood (with measured volume) were extracted from each mouse, weighed, and freeze-dried.

The gold content in the isolated tissues was determined using neutron activation analysis at the IBR-2 reactor in Dubna, Russia. The description of irradiation channels and pneumatic transport system of the REGATA installation can be found elsewhere [34]. 

The analyzed samples were packed in aluminum foil cups, irradiated for 3 days at a neutron flux 1.2 × 10^11^ cm^−2^ s^−1,^ and measured for 30 min after 4 days of irradiation. The samples were irradiated simultaneously with two reference materials: SRM 2710 (Montana Soil, Highly Elevated Trace Element Concentrations (NIST, USA) and the liquid gold standard (Merck, Darmstadt, Germany). Reference materials and blanks were placed in each container. 

Gamma spectra of induced activity were measured using spectrometers based on HPGe detectors with an efficiency of 40–55% and resolution of 1.8–2.0 keV for total-absorption peak 1332 keV of the isotope ^60^Co and Canberra spectrometric electronics. The spectra analysis was performed using the Genie2000 software (version 3.4.1) (Canberra Industries, Inc.; Toledo, OH, USA) and the software ‘‘Concentration’’ version 6.13.3 (JINR, Dubna, Russia). 

In the present study, with lower net inaccuracy the isotope 198Au from the reference material liquid standard was used as a calibrator, and SRM 2710 was used for quality control. The obtained values for concentrations for SRM 2710 in all irradiated containers differed from the certified values in the range (0.2–5.5%).

### 4.5. Behavioral Tests

*Assessment of anxiety levels.* Experimental (10 males, 10 females) and control (10 females) mice entered elevated Plus-maze (Open Science, Russia): basic test protocol for estimation of anxiety levels was applied [35], with only the trial duration taken from verification protocol given by the manufacturer of test installation (Verification of elevated Plus-maze by Open Science company’s scientific division [36] Test was done in one day for every 10 animals, with one test trial per animal; mice were not previously acquainted with the test. The plus-shaped construction consists of four arms, connected to the common center at 90 degrees angle, and it is elevated at 40 cm height above the table. Two arms are open walkways without walls, and the other two arms have walls from three sides, except the central side. Near the maze, two lamps were positioned opposite to each other from the open arms’ sides, so the pronounced contrast lighting of open and closed arms was created: open arms were bright, and closed arms were dark. 

Animals were consequently tested; before each trial floor and wall of all arms and centers were cleaned with ethanol to prevent smell marks’ influence on mice’s behavior. According to the protocol of preparation for this test [35], before the first animal’s trial one “zero” trial was done to equalize test conditions for the first mouse with others’, one mouse that was not included in this experiment was set in the test for 3 min without collection of data. The animal was placed in the maze center and then had 3 min to roam freely. After that the mouse was gently extracted from the maze and placed in its cage; the maze was cleaned with 95% ethanol before the placement of the next animal.

Video records of elevated plus-maze trials were analyzed in the RealTimer program (OpenScience, Moscow, Russia) (free distribution). Seven aspects of behavior were counted and measured; when a mouse was in closed arms, open arms, or center, running and rearing (with support—when forelegs were placed on the wall), grooming and the stretch-attend posture, when an animal stands with forelegs in one zone and hind legs in other zone and stretches its body to sniff and look. For each aspect four quantitative parameters were measured, including number of events, summary length (s), mean length (s), and latency to the first event (s).

*Assessment of spatial learning and memory*. One cycle of testing (three days of primary learning) was performed in the Morris water maze (MWM), according to the early developed protocol of the testing [37,38] with additional assessment of the flexibility of developed spatial memory by subsequent re-positioning of the platform into the other part of the swimming pool (fourth day) [39]. The circular white pool (1.5 m in diameter) was filled with water, and various visible clues were positioned around the pool. Water was made opaque with dry milk to hide the underwater platform. Each animal had to find this invisible underwater platform to escape from the water. If the animal did not find the platform during a trial, it was gently placed on the platform manually. 

The cycle consisted of four consecutive days with three daily trials: the platform’s location was unchanged in the first three days of primary learning, but on the fourth day, the platform was relocated to the other part of the pool for assessment of formed spatial memory. During each trial, a mouse was allowed to swim freely in the pool for a maximum of 180 s. When the animal either found the platform or was placed on it after the time was up, it was left to sit on the platform for 20 s, and then it was picked up, dried with a clean cloth, and returned to its cage until its next trial. Therefore, when 15 mice entered the test, each animal had 20–30 min of rest between the trials.

For each animal was determined the behavioral type was demonstrated during trials in three days of primary learning. Similarly to the previously developed protocol [40], the general sample was divided into three groups, “incapable”, “capable”, and “intermediate” animals, based on the type of behavior most frequently shown on Day 3. Incapable mice were excluded from the MWM as being non-responsive to potential changes induced by AuNPs that could be detected in this test; they demonstrated types of behavior, which did not include exploration of the pool. Therefore, they did not settle in the paradigm of MWM. All other individuals demonstrated various types of behavior that included an exploration of the pool, so they were assigned either to the capable (directed search, “scanning” [41] or to the intermediate (random search) group. 

Thus, 30 young experimental mice, 15 females and 15 males, entered MWM, and data from 24 animals were included in the statistical analysis, with both genders present in each group. After MWM the offspring aged out at 2 months; therefore, to minimize the influence of aging, the data from control female offspring (their mothers drank clear water) from the previous experiment with a similar design (with silver nanoparticles; [33]) were taken into the analysis as the control, and also the control sample included the data from female offspring from the previous experiment with a similar design in which titanium dioxide nanoparticles were too big to pass through the blood–brain barrier so titanium was not detected in the brain at all [42].

Video records of MWM trials were analyzed by the Ethovision program (Noldus Information Technology, Wageningen, The Netherlands). Trajectories (tracks) of all trials were visualized and based on those three parameters were calculated for each trial: the distance moved (track length (cm), the latency to the platform (s), and the total distance to the platform (cm). The last parameter is a sum of distances to the platform from all points of the track; if the value is low, this means that a mouse was swimming near the platform. Means of the parameters per day were calculated for each animal, with subsequent comparison of the daily samples of the means.

### 4.6. Statistics

The behavioral tests’ results were analyzed using Statistica 13.2 software (Dell Inc., Round Rock, TX, USA), at the significance level of *p* < 0.05; all tests were two-tailed. The elevated plus-maze results were compared between experimental and control animals for each parameter of each behavioral aspect by Mann–Whitney U test. Additionally, the results for experimental males or females were compared with the control group.

For MWM results distribution of quantitative variables was checked for normality by Shapiro–Wilk test; this hypothesis was rejected for all three parameters. The daily changes of all three parameters (learning curves) for each group in the experimental and control samples were assessed by comparison between Day 1 and Day 3 in Wilcoxon matched pairs test. Differences between the experimental and control data of each group were assessed by each day comparison with Mann–Whitney U-test.

## 5. Conclusions

Gold was present in all examined organs of experimental females and their offspring. The highest gold content was found in the kidneys, followed by the liver, which suggests that in case of prolonged exposure excretion of AuNPs was done by the kidneys. In contrast, the liver eliminated AuNPs from the bloodstream and accumulated them. The content of gold in the offspring’s liver and blood was lower than in the mother’s indicating a relatively low rate of AuNPs’ transfer to offspring. Accumulation of gold in the brain was the lowest among analyzed organs both in females and offspring, which could be attributed to the chemical inertness of gold. No significant differences in spatial orientation and memory were found between experimental and control offspring in MWM. Still, the experimental young mice demonstrated increased anxiety levels in an elevated Plus-shaped maze. Thus, the AuNPs influenced the emotional state of mice that were exposed to nanoparticles during prenatal and early postnatal development, but not on their cognitive abilities.

## Figures and Tables

**Table 1:** Content of gold in organs and tissues of experimental females and their offspring.

Sample	Organ or Tissue	Mean ± SD (μg/g Dry Weight)	Range (μg/g Dry Weight)
Females (5)	Blood	0.012 ± 0.007	0.006–0.023
	Liver	0.239 ± 0.117	0.131–0.410
	Kidneys	0.323 ± 0.128	0.182–0.516
	Lungs	0.079 ± 0.049	0.038–0.145
Offspring (10)	Blood	0.007 ± 0.005	0.002–0.018
	Liver	0.028 ± 0.022	0.008–0.083
	Kidneys	0.161 ± 0.116	0.053–0.418
	Lungs	0.017 ± 0.010	0.005–0.036

**Table 2:** Gold accumulated in the brain of female mice and their offspring, excluding its content in blood vessels in the brain.

Sample	Specific Mass in a Sample (ng)		Gold Content (ng/g Dry Weight)
	Mean ± SD	Range	
Females (5)	0.25 ± 0.10	0.14–0.36	3.79
Offspring (10)	0.08 ± 0.03	0.04–0.13	1.10

## Data Availability

All data are presented in Supplementary Files.

## Supplementary Materials

The following supporting information can be downloaded at: https://www.mdpi.com/article/10.3390/ijms24108962/s1.
