# Supplementary material for: Impact of Chronic Oral Administration of Gold Nanoparticles on Cognitive Abilities of Mice
Source: Int J Mol Sci. 2023 May 18;24(10):8962. doi: 10.3390/ijms24108962 (PMC10219544; doi:10.3390/ijms24108962)
Supplement: Supplementary file 1 [file ijms-24-08962-s001.zip › ijms-2381653-supplementary.pdf]

Supplement S1. Elevated Plus-shaped maze results

Table S1. Elevated Plus-shaped maze results – different zones of the maze (number of events, summary length)

Contact with AuNP		Closed arms		Open arms		Center	
		Number of events	Summary length, s	Number of events	Summary length, s	Number of events	Summary length, s
Control	Mean	4.6	123.275	0.9	11.599	6	45.703
	SD	2.6	42.0	1.3	18.3	3.5	29.7
	Median	4.5	132.115	0	0	5.5	44.33
	Q1	3	93.3	0	0	4	18.05
	Q3	6	160.99	2	25.85	9	76.38
	Min	1	45.9	0	0	1	6.37
	Max	9	172.43	3	47.08	12	87.02
Experiment	Mean	2.2	150.801	0.7	14.5	2.95	14.67
	SD	1.74	44.93	1.22	41.02	2.96	16.81
	Median	1	170.82	0	0	1.5	8.32
	Q1	1	151.38	0	0	1	5.42
	Q3	3.5	173.81	1	5.84	4	16.37
	Min	0	0	0	0	1	0.43
	Max	6	179.57	5	178.04	11	67.04

Table S2. Elevated Plus-shaped maze results – different zones of the maze (mean length, latency to the first event)

Contact with AuNP		Closed arms		Open arms		Center	
		Mean length, s	Latency to the first event, s	Mean length, s	Latency to the first event, s	Mean length, s	Latency to the first event, s
Control	Mean	46.415	26.94	4.637	11.943	10.716	9.774
	SD	48.6	19.9	6.5	26.4	10.9	11.9
	Median	29.41	26.37	0	0	7.315	4.895
	Q1	15.55	11.75	0	0	2.61	0.24
	Q3	45.54	38.25	12.65	5.4	12.43	15.67
	Min	9.81	1.12	0	0	1.59	0.09
	Max	160.99	63.39	15.69	83.32	38.19	38.25
Experiment	Mean	106.5	16.9	11.2	20.4	5.7	20.9
	SD	69.10	37.20	39.43	51.85	4.96	47.08
	Median	121.8	6.7	0	0	4.9	4.3
	Q1	37.7	3.8	0	0	2.6	2.3
	Q3	172.50	13.91	5.54	3.58	7.22	8.32
	Min	0	0	0	0	0.43	0.29
	Max	179.57	171.00	178.04	176.81	24.31	171.00

Table S3. Elevated Plus-shaped maze results – rearing and running (number of events, summary length)

Contact with AuNP		Rearing		Running	
		Number of events	Summary lenght, s	Number of events	Summary lenght, s
Control	Mean	8.6	8.146	34.2	23.698
	SD	6.6	7.5	10.4	8.8
	Median	5.5	4.405	39	23.725
	Q1	4	2.54	29	18.72
	Q3	15	13.25	41	30.07
	Min	0	0	11	6.51
	Max	18	19.63	45	38.17
Experiment	Mean	10.1	15.07	23.85	20.452
	SD	5.39	9.06	9.77	9.27
	Median	10	13.37	24	18.63
	Q1	7	9.88	16	13.72
	Q3	13	21.26	32	26.30
	Min	0	0	7	6.83
	Max	22	40.78	40	40.14

Table S4. Elevated Plus-shaped maze results – rearing and running (mean length, latency to the first event)

Contact with AuNP		Rearing		Running	
		Mean lenght, s	Latency to the first event, s	Mean lenght, s	Latency to the first event, s
Control	Mean	0.768	46.946	0.685	15.035
	SD	0.3	51.0	0.1	15.7
	Median	0.865	32.73	0.685	10.35
	Q1	0.61	7.97	0.6	2.7
	Q3	0.96	68.39	0.81	23.51
	Min	0	0	0.49	1.21
	Max	1.23	172.2	0.85	45.53
Experiment	Mean	1.4	22.3	0.9	6.6
	SD	0.51	23.01	0.29	7.34
	Median	1.4	15.0	0.8	4.1
	Q1	1.3	8.3	0.7	2.2
	Q3	1.70	30.93	1.03	7.53
	Min	0	0	0.49	0.40
	Max	2.39	96.15	1.73	28.57

Table S5. Elevated Plus-shaped maze results – stretch-attend posture and grooming (number of events, summary length)

Contact with AuNP		Stretch-attend posture		Grooming	
		Number of events	Summary lenght, s	Number of events	Summary lenght, s
Control	Mean	9.1	41.848	3.3	8.213
	SD	3.6	24.8	2.4	5.5
	Median	8.5	35.605	3	7.36
	Q1	7	27.99	2	4.27
	Q3	10	51.26	5	13.82
	Min	5	16.78	0	0
	Max	17	104.67	8	16.29
Experiment	Mean	5.6	21.231	2.3	18.59
	SD	3.47	15.37	1.59	21.69
	Median	5	20.41	2	11.22
	Q1	3	10.28	1	4.48
	Q3	8	28.99	3	24.22
	Min	0	0	0	0
	Max	12	50.88	7	91.87

Table S6. Elevated Plus-shaped maze results – stretch-attend posture and grooming (mean length, latency to the first event)

Contact with AuNP		Stretch-attend posture		Grooming	
		Mean lenght, s	Latency to the first event, s	Mean lenght, s	Latency to the first event, s
Control	Mean	4.99	18.095	2.658	76.198
	SD	3.2	20.1	1.9	50.1
	Median	4.245	12.215	2.69	80.005
	Q1	2.8	0.46	1.42	39
	Q3	5.85	33.36	2.95	93.92
	Min	2.1	0.13	0	0
	Max	13.08	63.25	7.06	161.03
Experiment	Mean	3.4	18.4	7.5	89.0
	SD	2.16	29.49	7.03	47.88
	Median	3.7	6.3	4.7	98.6
	Q1	1.8	2.2	3.1	49.0
	Q3	5.47	27.02	9.00	123.75
	Min	0	0	0	0
	Max	6.70	126.17	25.53	176.17
